# Effects of the Presence and Behavior of In-Group and Out-Group Strangers on Moral Hypocrisy

**DOI:** 10.3389/fpsyg.2020.551625

**Published:** 2020-09-15

**Authors:** Junfeng Bian, Liang Li, Xuan Xia, Xiaolan Fu

**Affiliations:** ^1^Research Center of Social Governance Innovation, Changsha University of Science and Technology, Changsha, China; ^2^Institute of Psychology, Chinese Academy of Sciences (CAS), Beijing, China; ^3^Department of Psychology, University of Chinese Academy of Sciences, Beijing, China; ^4^Hunan Provincial Bureau of Statistics, Changsha, China; ^5^Hunan University of Technology and Business, Changsha, China

**Keywords:** moral hypocrisy, in-group, out-group, moral behavior, hypocritical behavior

## Abstract

Moral hypocrisy (MH) occurs when people fail to practice what they preach. Despite the prevalence of the effect of social identity on an individual’s MH, few empirical studies have explored contextual factors that may help reduce MH. By conducting two experiments based on the research paradigm of real stranger presence, we examined how in-group and out-group strangers’ presence and moral behavior may contribute to reducing MH. The results of experiment 1 demonstrated that compared with the presence of out-group strangers, the presence of in-group strangers could effectively inhibit MH (no significant difference between participants reported and actual donation proportions was obtained). The results of experiment 2 replicated and extended the results of experiment 1, first by showing that the presence of in-group strangers could effectively inhibit MH and then by revealing the influence of present strangers’ behavior (moral or hypocritical) on MH. The results indicated that strangers’ moral behavior could effectively eliminate participants’ MH, especially in the presence of in-group strangers. However, when present strangers exhibited hypocritical behavior, they exhibited no effect on participants’ MH, irrespective of the condition of in-group and out-group strangers. The current study provides empirical support for theories related to MH and moral decision-making and contributes to the literature on in-group and out-group effects on MH and decision-making.

“Hypocrisy is the homage vice pays to virtue.”

—François, duc de la Rochefoucauld

## Introduction

Throughout history and daily life, examples can be found of moral people who often fail to act morally. For example, a news agency exposed a recognized national model of morality for engaging in criminal and unethical business behavior (Alicke et al., 2013; Rossi, 2018). Prominent research questions include why people take a public stand against crimes they have previously committed and why moral people behave in an immoral manner. Studies have revealed the nature of moral behavior to be hypocrisy; that is, people desire to appear moral to themselves and others without practicing moral behavior (Batson et al., 1997, 1999, 2002).

Moral hypocrisy (MH) is the desire to behave morally while seeking opportunities to avoid adopting behaviors that actually result in morally good outcomes (Batson et al., 1997). MH has two manifestations. First, people adopt harsher and stricter standards of moral judgment for others than those they adopt for themselves (Valdesolo and DeSteno, 2008; Lammers et al., 2010), and this type of hypocrisy occurs at the intrapersonal level. Second, at the interpersonal level, hypocrisy is manifested in people’s morality standards, which are inconsistent with their actual behavior; that is, their actual behavior fails to meet their claimed moral requirements of behavior (Watson and Sheikh, 2008; O’Connor et al., 2020). Batson et al. (1997) investigated hypocrisy and explained the cause for moral people apparently behaving immorally. They indicated that self-interest often overrides the goal of behaving with integrity, leading to hypocrisy. People might be sensitive to hypocrisy resulting from the deliberate pursuit of self-serving goals. A groundbreaking series of studies carried out by Batson and colleagues disclosed the commonness of MH, the motivation to appear moral yet, if possible, avoid the cost of actually behaving morally (Batson et al., 1997, 1999, 2002; Wagner et al., 2019). Moreover, on the basis of this finding, researchers discussed factors that influence people’s MH, such as individual anger (Polman and Ruttan, 2011; Laurent et al., 2014), power (Lammers et al., 2010; Rustichini and Villeval, 2014), and conformity values (Lönnqvist et al., 2014). By contrast, guilt and religious beliefs (Polman and Ruttan, 2011) and awareness (Aronson et al., 1991; Stone et al., 1994; Batson et al., 2002) inhibit MH. By investigating MH at the interpersonal level, Lönnqvist et al. (2014) deduced that MH is the process of impression management or self-deception. Hypocritical people not only adopt self-interest behavior but also desire to maintain a virtuous moral image (Caviola and Faulmüller, 2014; Lönnqvist et al., 2014; Chance et al., 2015; Tang et al., 2018). Two types of explanations have been provided for hypocrisy: social preference explanations (Fehr and Schmidt, 1999) and social signaling explanations (Barden et al., 2014). Most studies have demonstrated the influence of MH at the intrapersonal level (Lammers et al., 2010; Polman and Ruttan, 2011; Laurent et al., 2014; Lönnqvist et al., 2014; Rustichini and Villeval, 2014; Lindenberg et al., 2018; Wojtek, 2019), and the factors that influence the interpersonal level have rarely been discussed (Fu et al., 2015). Despite the prevalence of the influence of social identity on people’s MH, few empirical studies have explored the contextual factors that may help reduce MH. MH remains an interesting but inconclusively supported behavior hypothesis highly influenced by interpersonal factors.

Studies have revealed that people often have higher tolerance for violations of moral principles performed by themselves or in-group members because their opinions regarding moral principles are different for themselves or in-group members for immoral behaviors adopted toward others (Valdesolo and DeSteno, 2008). According to social influence theory, the presence of others, immediacy, and strength affect the actual behavior of people during interactions (Latané, 1981). People are aware of the possibility that they may leave the impression of being immoral on others who observe a discrepancy between their actual behavior and a declared moral level; therefore, under the pressure of impression management, an individual may inhibit MH in front of others. The status of onlooking strangers as in-group or out-group members influences the effects on MH. Research addressing the question of whether people’s moral behavior is influenced by the in-group/out-group distinction has indicated that the presence of an ordinary stranger does not inhibit MH (Barden et al., 2014; Fu et al., 2015). When people encounter strangers who are not influential or only on one occasion, they may place less importance on the adoption of behavior. However, it is unclear whether MH is inhibited by the presence of a stranger who is an in-group member. Accordingly, we propose **hypothesis 1: the presence of an in-group stranger effectively inhibits MH, whereas that of an out-group stranger does not**.

Fu et al. (2015) indicated that in a particular situation, strangers are not merely observers; rather, they usually exhibit specific behaviors. Social learning theory suggests that the ambiguity of social situations tends to cause individuals feelings of uncertainty (such as regarding whether others would adopt moral behavior in their situation) and that the most direct method of overcoming this uncertainty is through the observation of others. It is unclear whether a demonstration of moral or hypocritical behavior by a stranger of a different social identity influences people’s MH. Moreover, according to Festinger’s (1957) classic theory of cognitive dissonance, when an individual engages in a behavior contrary to their attitude, they must look for an excuse to relieve the dissonance they experience. Thus, hypocrites must deceive not only others but also themselves; that is, the purpose of hypocrisy is to provide convincing evidence of morality (Batson et al., 1999, 2002). When moral standards are salient, it is difficult for people to avoid comparing their behavior to ethical standards, thus inhibiting MH. In addition, others with moral behavior may become role models for people, thereby inhibiting MH. By contrast, when others demonstrate hypocritical behavior, no salient moral standards are available and their demonstration may set a substandard example for others (Bandura, 1990), providing them with excuses for moral disengagement (Kish-Gephart et al., 2014). Therefore, we propose **hypothesis 2: The presence and behavior of in-group and out-group strangers may affect an individual’s hypocrisy; the moral behaviors of in-group strangers may inhibit MH, and their hypocritical behavior may induce hypocrisy.** Relevant experiments have used the difference between individuals’ real and reported donation amounts as a measure of their MH (Polman and Ruttan, 2011; Fu et al., 2015). Thus, to verify the aforementioned two hypotheses, the effect of the presence and behavior of in-group and out-group strangers on MH was investigated in current study through one pre-experiment and two formal experiments.

In a preliminary study, we determined whether the expected donation amount is affected by the total amount in the individual’s possession to validate the MH measure. Experiment 1 focused on the effect of the presence of strangers (in-group vs. out-group) on MH in donation situations, and experiment 2 focused on the effect of strangers’ behavior (moral vs. hypocritical) on MH.

### Preliminary Study

In the preliminary study, we developed a scenario-based questionnaire to determine whether the total amount individuals had would influence their donation amount. A total of 81 participants (39 male, *M*_*age*_ = 22.03, *SD* = 2.14) were recruited, and all were asked how much they would donate if they had 20 or 50 RMB^1^. Before the experiment, each participant signed an informed consent form, and each was paid after the experiment. The experiment was approved by the departmental ethics committee.

The results of preliminary analyses indicated no significant difference in the donation proportions between the two conditions (50 RMB: *M* = 0.47, *SD* = 0.35; 20 RMB: *M* = 0.48, *SD* = 0.29; *t* = 0.14, *df* = 80, *p* = 0.88). Therefore, we created an MH indicator by subtracting the proportion donated by participants (20 RMB in total) from the proportion expected (50 RMB in total). To prevent participants from guessing the research purpose, we set the total amount that the participants were expected to donate to 50 RMB, and the actual amount donated was set to 20 RMB in the formal experiment.

## Experiment 1

### Materials and Methods

#### Participants

Participants were 93 healthy college students (*M*_*age*_ = 20.73 years, aged 16–26 years, *SD* = 2.16; 54% female) who were randomly selected through a network. The participants were right handed with normal or corrected vision and no history of neurological or psychiatric disorders. After the experiment, researchers paid the participants. Before tests, each participant signed an informed consent form. A *post-hoc* power analysis using G^∗^Power (Faul et al., 2007) suggested that we had 99.26% power to detect the obtained interaction. The experiment was approved by the departmental ethics committee.

#### Materials

*Donation scenario materials*: According to the standard experimental paradigm, scenario-based materials have often been used in MH studies (Polman and Ruttan, 2011; Fu et al., 2015). Thus, two donation scenarios (hypothetical and real situations) were used (see Appendix).

#### Procedure

The experiment was conducted in two stages. First, each participant completed Tajfel’s pot estimation task^2^. Subsequently, they were randomly presented with “in-group stranger” and “out-group stranger” conditions (strangers were false participants). Next, the experimenter^3^ presented participants with the *donation scenario materials* for the hypothetical situation and asked them to report the amount they would donate. Participants were asked to complete some simple arithmetic questions (5 min). Afterward, the experimenter gave each participant 20 RMB.

Second, the experimenter presented participants with the *donation scenario materials* for the real situation along with the following prompt: “Suppose you are passing a donation site on your way to a lab. We want you to take this opportunity to help teachers. You can donate and write a message for the teacher, and we will forward your donation or message to the teacher.” After the experiment, the experimenter asked participants “How realistic do you think this donation scenario is?” Finally, the experimenter explained the experiment purpose to participants.

### Results

Nine participants were excluded because they considered the donation situation to be unrealistic. We compared the expected and actual amount donated by participants assigned to the two conditions (the presence of in-group and out-group strangers) by using paired-sample *t*-tests, and the differences in donation proportion values between the pre-donation (reported donation) and post-donation (actual donation) scenarios were compared through an independent *t*-test.

#### The Amount and Proportion of Participants’ Reported and Actual Donation

Tables 1, 2 present the descriptive statistics of the donation amount reported by participants under the two experimental conditions, the actual donation amount, and the proportion of reported and actual donation amounts.

**TABLE 1:** The descriptive statistics of the amount of individual reported and actual donated in two conditions.

**Experimental condition**		**Report donation amount**	**Actual donation amount**
			
	***n***	***M* ± *SD***	***M* ± *SD***
Presence of in-group stranger	41	31.68 ± 12.52	11.66 ± 3.55
Presence of out-group stranger	43	34.02 ± 14.91	6.11 ± 4.78

**TABLE 2:** The descriptive statistics of the proportion of individual reported and actual donated in two conditions.

**Experimental condition**		**Report donation proportion**	**Actual donation proportion**
			
	***n***	***M* ± *SD***	***M* ± *SD***
Presence of in-group stranger	41	0.65 ± 0.26	0.59 ± 0.18
Presence of out-group stranger	43	0.69 ± 0.39	0.32 ± 0.24

Moreover, for each condition, we compared the proportion that participants reported that they would donate with the proportion that they actually donated (Table 3).

**TABLE 3:** The comparison of the proportion of individual reported and actual donated in two conditions.

**Experimental condition**	***n***	***t***	***p***
Presence of in-group stranger	41	1.16	0.26
Presence of out-group stranger	43	3.43	0.00

Table 3 reveals no significant difference between the reported donation proportion and the actual donation proportion among participants under the in-group stranger condition (*p* > 0.05). By contrast, the actual donation proportion was significantly lower than the reported donation proportion among participants under the out-group stranger condition (*p* < 0.001).

#### Influence of In-Group/Out-Group Strangers’ Presence on MH

To investigate the influence of the presence of in-group/out-group strangers on MH, we compared the two groups with respect to the difference between reported and actual donation amounts by using an independent *t*-test (Table 4).

**TABLE 4:** The comparison of the difference between the proportion of individual reported and actual donated in two conditions.

**Experimental condition**	**The difference between the proportion of reported and actual donated**		
	***M* ± *SD***	***t***	***p***
Presence of in-group stranger	0.06 ± 0.31	−2.95	0.01
Presence of out-group stranger	0.37 ± 0.58		

The results indicated a significant difference (*p* < 0.05) between reported and actual donation proportions under both the condition of the presence of in-group strangers (*p* = 0.05; *SD* = 0.24) and the condition of the presence of out-group strangers (*p* = 0.36; *SD* = 0.58).

Research (Fu et al., 2015) has indicated that strangers’ moral behavior inhibits people’s MH. When the stranger present is not simply a bystander but also demonstrates moral or hypocritical behavior (declares willingness to donate and the subsequent behavior is consistent or inconsistent) and provides a behavior reference for people, this makes the moral standards more salient, and then how does this effect participant’s MH? Therefore, Experiment 2 was conducted to investigate the influence of the presence and behavior of in-group and out-group strangers on the MH.

## Experiment 2

### Materials and Methods

#### Participants

Participants were 164 healthy college students (*M*_*age*_ = 21.14 years, aged 17–25 years, *SD* = 1.82; 65% female) who were randomly recruited. According to their answers, six participants were rejected (those who considered the donation scenario to be unrealistic). The participants were right handed with normal or corrected vision and no history of neurological or psychiatric disorders. After the experiment, the participants were paid. Each participant signed an informed consent form. A *post-hoc* power analysis using G^∗^Power suggested that we had 99.42% power to detect the obtained interaction. The experiment was approved by the departmental ethics committee.

#### Materials

*Donation scenario materials*: the same as those used in experiment 1.

*Other materials*: a donation box, the introduction material of recipients, and some envelopes.

#### Procedure

False participants entered the laboratory early. When the real participants entered, the false participants greeted them with a sincere “hello.” The false and actual participants did not communicate during the subsequent experiment.

First, similar to experiment 1, participants in this experiment were assigned to the “in-group stranger” or “out-group stranger” condition (strangers were false participants). The experimenter showed participants the hypothetical version of the *donation scenario materials* and asked them to report the amount they would donate (first, the false participant reported that they were willing to donate 50 RMB). Subsequently, both the false and actual participants completed simple arithmetic questions (5 min) and were paid 20 RMB.

Second, the real donation situation was explained to the participants, in the same manner as in experiment 1, and they were asked to place the donation amount in an envelope. The false participants, who were arranged to be in the moral behavior group that donated first, took 20 RMB yuan from the envelope and placed the amount in the donation box, and the false participants of the hypocrisy group neither donated money nor wrote a message. After the experiment, the experimenter asked participants “How realistic do you think this donation scenario is?” Finally, the experimenter explained the real purpose of the experiment to the participants.

### Results

We compared the reported and actual donation amounts of participants under two conditions (presence of in-group and out-group strangers, moral and hypocritical behaviors) by conducting a paired-sample *t*-test and independent sample *t*-test between groups (presence of in-group and out-group stranger, moral and hypocritical behaviors). Finally, a 2 (experiment condition: in-group vs. out-group stranger) × 2 (behavior: moral vs. hypocritical) analysis of variance (ANOVA) was performed with gender as a covariate. The dependent variable was the proportion of the difference between reported and actual donation amounts.

#### Amount and Proportion of the Reported and Actual Donation Amounts of Participants

Tables 5, 6 present the descriptive statistics of the proportion of reported and actual amounts donated by the participants under the four conditions (the presence of in-group/out-group strangers, strangers’ moral/hypocritical behavior).

**TABLE 5:** The descriptive statistics of the amount of the individual reported and actual donated in four conditions.

**Experimental condition**		**Report donation amount**	**Actual donation amount**
	***n***	***M* ± *SD***	***M* ± *SD***
Presence of in-group stranger	79	36.68 ± 12.52	14.66 ± 3.55
Presence of out-group stranger	79	42.02 ± 14.91	11.75 ± 4.78
Moral behavior	79	45.14 ± 11.62	16.55 ± 6.11
Hypocritical behavior	79	36.77 ± 12.07	9.88 ± 6.98

**TABLE 6:** The descriptive statistics of the proportion of individual reported and actual donated in four conditions.

**Experimental condition**		**Report donation proportion**	**Actual donation proportion**
	***n***	***M* ± *SD***	***M* ± *SD***
Presence of in-group stranger	79	0.71 ± 0.26	0.68 ± 0.18
Presence of out-group stranger	79	0.83 ± 0.39	0.63 ± 0.24
Moral behavior	79	0.86 ± 0.21	0.85 ± 0.30
Hypocritical behavior	79	0.73 ± 0.27	0.51 ± 0.34

Furthermore, we compared the proportions of the difference between the reported and actual donation amounts of participants under the four conditions. The results revealed that the difference in proportion was significantly greater under the condition of the presence of out-group strangers than under the condition of the presence of in-group strangers (*p* < 0.01). The presence of strangers from an in-group caused an effective inhibition of participants’ hypocritical behavior, and that of out-group strangers did not. This result is consistent with that of study 1. Moreover, when the stranger exhibited moral and hypocritical behaviors, the difference between reported and actual donations was 0.01 (*SD* = 0.31) and 0.22 (*SD* = 0.42), respectively. When the stranger exhibited hypocritical behavior, the difference between reported and actual donation amounts was significantly higher than that when the stranger exhibited moral behavior (*p* < 0.001). The moral behavior of the stranger effectively inhibited participants’ MH, and the hypocritical behavior of the stranger did not.

#### Influence of the Presence and Behavior of In-Group/Out-Group Strangers on MH

To examine the influence of the presence and behavior of in-group/out-group strangers on MH, the identity of strangers and their behavior were assessed through ANOVA. The results indicated that the main effect of identity of strangers was significant (*F* = 9.31, *p* < 0.01). Under the condition of the presence of out-group strangers, the difference between reported and actual donations was significantly higher than that under the presence of in-group strangers. Moreover, the main effect of the stranger’s behavior was significant (*F* = 6.62, *p* < 0.05). When the stranger exhibited hypocritical behavior, the difference between reported and actual donations was significantly higher than when the stranger exhibited moral behavior (*F* = 4.40, *p* < 0.05). The identity and behavior of the stranger exerted significant interactive effects on the participants’ behavior. The results of a further simple effect analysis revealed that under the in-group and out-group stranger conditions, when the stranger behaved morally, no significant difference between the reported and actual donation proportion was obtained (*F* = 0.39, *p* > 0.05). By contrast, when the stranger exhibited MH, a significant difference between the reported and actual donation proportion was noted under both in-group and out-group stranger conditions (*F* = 0.39, *p* > 0.05). In addition, no significant difference between the reported and actual donation proportion (*F* = 1.48, *p* > 0.05) was obtained under the condition of in-group stranger presence, regardless of whether the stranger exhibited moral or hypocritical behavior. A significant difference between the reported and actual donation proportion (*F* = 8.99, *p* < 0.05) was obtained under the condition of the presence of out-group strangers, irrespective of whether strangers exhibited moral or hypocritical behavior.

## Discussion

In this study, the influence of the presence and moral or hypocritical behavior of strangers on participants’ MH was examined using scenario-based experimental paradigms, which are widely used to assess moral behavior (Fernandez-Dols et al., 2010; Bian et al., 2019; Tillmann et al., 2019). As expected, the presence and moral behavior of in-group strangers exhibited a measurable effect on participants’ MH, including the ability to inhibit their hypocritical behavior. When a stranger behaved morally, the presence of the in-group members could inhibit an individual’s hypocritical behavior, and the presence and hypocritical behavior of out-group members did not inhibit individuals’ hypocritical behavior.

### Effects of the Presence of In-Group or Out-Group Strangers on MH

The results of experiment 1 demonstrated that the presence of in-group strangers could effectively inhibit participants’ MH and that the presence of out-group strangers did not inhibit participants’ MH. Furthermore, studies have reported that such identity information affects people’s moral decisions (Kelman, 1958; Caviola and Faulmüller, 2014; Lönnqvist et al., 2014; Carlos and Lewis, 2018). To maintain a virtuous moral image, people may refrain from adopting hypocritical behaviors (Fu et al., 2015; Jojanneke et al., 2015). Moreover, people who were grouped with the in-group stranger cared more about their reputation in the group and took considerable measures for impression management. Compared with the presence of out-group strangers, the presence of in-group members was of greater concern to the participants and promoted more prosocial behavior (Reinders Folmer and De Cremer, 2012; Liu and Lin, 2018). By contrast, individuals exhibited MH when confronted with the presence of out-group strangers. According to social influence theory, an individual’s attitudes and subsequent actions or behaviors are influenced by referent others; however, this influence depends on how important these other people are to the individual (Tong and Yang, 2011; Effron et al., 2018; Linden et al., 2019). Thus, people attempt to appear moral while avoiding the cost of actually adopting moral behaviors in the presence of out-group strangers.

### Effects of the Behavior of In-Group or Out-Group Strangers on MH

Furthermore, the results of experiment 2 demonstrated that the presence and moral behavior of in-group strangers could inhibit MH, whereas the presence of out-group strangers did not have this inhibitory effect. These findings are consistent with those of previous reports (Fu et al., 2015) and suggest that people may attempt to avoid incurring the cost of actually adopting moral behaviors, highlighting the value of social impression management. In particular, participants under the condition of the presence of out-group strangers were prone to adopting hypocritical behavior (Batson et al., 1997, 1999, 2002).

These findings have three possible explanations. First, cognitive dissonance explanations suggest that psychological conflict results from simultaneously held incongruous beliefs and attitudes. In this case, MH is a primary strategy for reducing the discomfort of this conflict (Reinders Folmer and De Cremer, 2012; Dong et al., 2019). People often feel uncertain in ambiguous situations; generally, their behavior depends on different social requirements. When in such a situation, the most convenient behavior for people to adopt is the behavior of others; this enables them to obtain useful information for future reference as well as demonstrate virtuous behavior to improve their self-image. Therefore, people’s behavior often exhibits a positive effect on others. Furthermore, studies have reported that people’s moral identity is threatened when moral standards conflict with self-interest (Valdesolo and Desteno, 2007; Monin et al., 2009; O’Connor et al., 2020). To reduce cognitive conflict, people often adopt different approaches: when encountering moral standards, people usually avoid MH behaviors; when these behaviors are not avoided, they lower their moral standards to rationalize their hypocritical behavior (López-Pérez and Spiegelman, 2013; Kish-Gephart et al., 2014; Abdai and Miklósi, 2016). For this study, the moral behavior of strangers was undoubtedly the established moral standard. To reduce cognitive conflict, participants maintained consistency between their own behavior and moral standards and avoided hypocrisy by donating the actual amount they reported. Second, according to social learning theory, people disobey moral rules or frequently exhibit hypocrisy mainly because of insufficient learning from moral examples. Strangers who behaved morally set an excellent example for the participants, who used it as a reference and attempted to emulate and imitate these strangers’ behavior (Bandura, 1990). When strangers exhibited hypocritical behavior, moral standards were not prominent in the context. Hypocrisy may set a negative example for others, enabling people to avoid the conflict between self-interest and moral standards and threats to their moral identity, thereby resulting in moral disengagement and hypocritical behavior. In other words, unacceptable behavior can be adopted without self-censure (Houwing and Bussey, 2017; Runions et al., 2019). Finally, in accordance with evolutionary theory, studies have revealed that people clearly differentiate between in-group and out-group members in terms of assistance and reciprocity. For example, Graham et al. (2015) demonstrated that social orientation influences people’s performance in terms of in-group contribution. Compared with the presence of out-group strangers, the presence of in-group strangers prompted participants to behave more morally and donate more. An alternative explanation for this result could be that when the in-group stranger was present, participants considered the possibility of future profits, and accepting the inconsistency between moral standards and their own behavior was difficult. Moreover, deceiving others was not possible; thus, the participants tried to inhibit MH as much as possible to maintain consistency between their behavior and moral standards.

In addition, the self-categorization theory identifies three cognitive processes relevant to people being part of an in-group or out-group. Social identification is a process wherein an individual or another person or group of people identifies with an in-group more overtly. The norms and attitudes of others within a group are considered compatible with those of an individual and worthy of emulation (Tajfel, 1986; Stellar and Willer, 2018; Wojtek, 2019). Therefore, an increase in participants’ MH in the presence of out-group strangers caused by the strangers’ social identity could be a plausible but tentative interpretation of the results because more empathetic people were included based on our preselection procedure for experiment. Thus, compared with the condition of the presence of out-group strangers, the condition of presence and moral behavior of in-group strangers exhibited a higher influence on participants’ moral behavior. When an in-group stranger behaved morally, participants may agree with it and remain consistent with the stranger’s behavior, avoid MH, and donate large amounts. Moreover, the results suggest that behavioral ethics research must employ the measures of actual behavior rather than hypothetical behavior. The average decision was affected by whether the behavior was real or hypothetical (Lönnqvist et al., 2011).

### Limitations and Future Research Direction

First, the experiments did not consider socio-demographic factors such as self-esteem and socioeconomic status, which may affect people’s perception of social prestige and impression management and directly affect their decision-making, future studies may examine self-esteem and socioeconomic status and test their respective contributions to the MH. Second, the present study did not investigate participants’ cultural values on MH. Culture impacts the ways in which people communicate as well as the strategies they use to communicate; it is a crucial moderator in explanation mechanism of human behavior (Rossi, 2018). Future studies may examine different cultural values to obtain more evidence for the distinct effects of MH.

## Conclusion

The current study demonstrated that (a) the presence of in-group strangers could effectively inhibit MH and (b) the moral behavior of in-group strangers could effectively inhibit participants’ MH. However, when present strangers exhibited hypocritical behavior, they had no effect on participants’ MH, irrespective of the condition of in-group or out-group status. These findings provide empirical support for theories related to MH and moral decision-making and contribute to the literature on in-group and out-group effects on MH and decision-making.

## Data Availability Statement

All datasets generated for this study are included in the article/supplementary material.

## Ethics Statement

The studies involving human participants were reviewed and approved by the Changsha University of Science and Technology. The patients/participants provided their written informed consent to participate in this study.

## Author Contributions

JB and XF conceived and designed the experiments and wrote the manuscript. JB, XX, and LL performed the experiments. JB analyzed the data. JB and XX contributed to the reagents, materials, and analysis tools. All authors contributed to the article and approved the submitted version.

## Conflict of Interest

The authors declare that the research was conducted in the absence of any commercial or financial relationships that could be construed as a potential conflict of interest.

## Footnotes

1 Before the preliminary study, we discovered that the amount that most people were willing to donate was 20 and 50 Yuan through an open-ended survey.

2 Because this is a false task with the only purpose of allowing the participants to form psychological common characteristics, the group list was determined using a main tester before the test.

3 In the experiment, male and female experimenters were arranged according to the participant gender.

## Appendix

### Authentic Situation

You are passing through a fundraiser. Grantee called Wen Han, male, 59 years old, and comes from Furong District, Hunan Province. He took root for 38 years in remote rural township, 28 years at the village primary school. Mr. Wen was praised as the loveliest teacher in Hunan Province. In July 2019, He was diagnosed with advanced lung cancer. During the next month, he spent all his savings on treatment. The monthly medical expenses (except medical insurance) are 15,000 yuan, a total cost of medical expenses more than 50 million. Mr. Wen is driven into a corner, and could do nothing about. Some people started the fundraiser and want to help the teacher Wen.

If you have 20 RMB yuan, how much do you want to donate to Mr. Wen?

### Hypothetical Situation

If a teacher is suffering from stomach cancer, treating the disease has spent all his savings, and now he urgently needs help.

If you have 50 RMB yuan, how much do you want to donate to Mr. Wen?
